# Supplementary material for: Reference-based compression of short-read sequences using path encoding
Source: Bioinformatics. 2015 Feb 2;31(12):1920–8. doi: 10.1093/bioinformatics/btv071 (PMC4481695; doi:10.1093/bioinformatics/btv071)
Supplement: Supplementary Data [file supp_31_12_1920__index.html]

Reference-based compression of short-read sequences using path encoding — Reference-based compression of short-read sequences using path encoding — Reference-based compression of short-read sequences using path encoding — Supplementary Data 

# Reference-based compression of short-read sequences using path encoding

## Supplementary Data

files

**Files in this Data Supplement:**

- Supplementary Data - pdf file
