# Supplementary material for: Reference-based compression of short-read sequences using path encoding
Source: Bioinformatics. 2015 Feb 2;31(12):1920–8. doi: 10.1093/bioinformatics/btv071 (PMC4481695; doi:10.1093/bioinformatics/btv071)
Supplement: Supplementary Data [file supp_btv071_Kingsford-Patro-supp.pdf]

Supplementary Material For:
Reference-based compression of short-read sequences using path encoding

Carl Kingsford and Rob Patro

1. Running times

All times and memory usages in these supplementary tables were obtained on a shared computer with 16 Intel Xeon 2.60GHz CPUs with 256Gb of RAM. SCALCE, fastqz, and PathEncode (version 0.6.3) were run as described in the main text. "PE User & Sys" gives the single-threaded time spent with system and user-level code. The time for CRAM is broken into alignment/samtools indexing and the actual compression.

Supplementary Table 1. Detailed running times for compressing the test files (in seconds).

Data	PathEncode	PE User & Sys	SCALCE	Fastqz	CRAM[a]
SRR037452	361	581	42	170	497 = 280 + 217
SRR445718	1344	2127	251	1161	1095 = 395 + 700
SRR490961	1979	3209	371	1750	1509 = 462 +1047
SRR635193	1250	1977	240	1244	1411 = 604 + 807
SRR1294122	1751	2795	294	1404	1218 = 447 + 771
SRR689233	1200	1752	208	1178	1464 = 373 + 1091
SRR519063	939	1398	413	1070	1602 = 346 + 1256

[a] CRAM running times are in the format total = (cram) + (bowtie & samtools).

Supplementary Table 2. Detailed running times for decompressing the test files (in seconds).

Data	PathEncode	PE User & Sys	SCALCE	Fastqz	CRAM
SRR037452	201	346	26	201	155
SRR445718	1185	1437	172	1168	270
SRR490961	1619	1933	277	1757	343
SRR635193	1135	1304	121	1279	398
SRR1294122	1417	1728	221	1356	326
SRR689233	1212	1351	118	1199	303
SRR519063	797	860	113	1187	284

2. Memory usage

The memory usage of each of the commands was measured using the `rusage` system call. All numbers are in kilobytes.

Supplementary Table 3. Memory usage for compressing the test files (in kB).

Data	PathEncode	SCALCE	Fastqz	CRAM
SRR037452	6,674,692	2,179,216	1,393,936	10,728,200
SRR445718	16,894,448	5,395,132	1,518,884	10,516,800
SRR490961	23,815,172	5,390,896	1,563,904	10,531,092
SRR635193	14,421,252	5,405,492	1,387,796	10,538,524
SRR1294122	19,548,580	5,395,184	1,467,556	10,522,252
SRR689233	13,338,780	5,368,152	1,494,000	10,538,344
SRR519063	13,792,636	5,356,136	1,563,904	10,530,808

Supplementary Table 4. Memory usage for decompressing the test files (in kB).

Data	PathEncode	SCALCE	Fastqz	CRAM
SRR037452	9,635,088	1,066,732	1,394,144	11,950,456
SRR445718	16,419,684	1,066,140	1,519,088	11,547,352
SRR490961	16,156,764	1,066,684	1,564,112	11,272,636
SRR635193	13,786,892	1,065,316	1,387,852	11,899,680
SRR1294122	16,677,436	1,067,248	1,485,488	11,722,704
SRR689233	14,509,168	1,067,580	1,494,228	10,804,052
SRR519063	9,431,372	1,066,032	1,564,116	10,619,432

3. Larger memory variant

Our implementation includes an alternative data structure to maintain the kmer counts. This variant uses more memory, but is faster (particularly for decompression). This mode, which produces the same compression as the standard mode, is suitable for use on large-memory machines.

Supplementary Table 5. Times and memory usage for the larger-memory implementation.

	Compression		Decompression	
Data	Time (s)	Memory (kb)	Time (s)	Memory (kb)
SRR037452	312	19,106,296	131	25,174,288
SRR445718	1106	23,236,324	735	28,475,596
SRR490961	1568	25,674,868	1016	31,700,676
SRR635193	919	21,434,852	616	27,182,760
SRR1294122	1349	25,008,828	874	30,094,116
SRR689233	820	22,890,932	640	25,854,564
SRR519063	708	21,854,032	490	25,679,332

4. Effect of observation multiplier on compression

Path encoding by default updates counts of transitions using a multiplier of $m = 10$ (Equation 2 in the main text). Using the `-mul` option, this can be changed to experiment with other weights. The effect of larger m values is to “forget” the initial reference counts more quickly (but errors seen multiple times will have a larger impact).

Supplementary Table 6. Effect of observation multiplier on compression.

Data set	Size $m = 10$	Size $m = 20$
SRR037452	43,105,624	43,415,969
SRR445718	154,960,810	156,530,538
SRR490961	170,613,303	171,548,006
SRR635193	187,256,974	190,117,465
SRR1294122	187,808,066	188,908,923
SRR689233	167,659,551	171,062,836
SRR519063	84,642,682	85,405,469
